# Supplementary material for: Discordant Health Implications and Molecular Mechanisms of Vitamin D in Clinical and Preclinical Studies of Prostate Cancer: A Critical Appraisal of the Literature Data
Source: Int J Mol Sci. 2024 May 13;25(10):5286. doi: 10.3390/ijms25105286 (PMC11120741; doi:10.3390/ijms25105286)
Supplement: Supplementary file 1 [file ijms-25-05286-s001.zip › ijms-2995623-supplementary.pdf]

Supplementary Materials to

Discordant Health Implications and Molecular Mechanisms of Vitamin D in Clinical and Preclinical Studies of Prostate Cancer: A Critical Appraisal of Literature Data

Annika Fendler, Carsten Stephan, Bernhard Ralla, and Klaus Jung

Supplementary Information: TCGA data used in Figure 2.

Gene expression data from the TCGA and GTEX cohorts were downloaded from UCSC Xena (https://xena.ucsc.edu/, accessed on 28 March 2024). Xena provides a dataset of ~20.000 RNA-seq datasets which were reanalysed using Toil to provide a batch-corrected dataset for meta-analysis [42,43] In brief, STAR was used for alignment to the Hg38 reference genome, RSEM, and Kallisto for count quantification. Finally, data were normalised in DeSeq2. After downloading the dataset, we extracted DeSeq2-normalised counts for VDR, LPR2, CYP24A1, CYP27B1, CYP2R1, and CYP27A1 for 646 prostate samples, including samples from both tumour and normal tissue (GTEX: 100 samples, TCGA: 546 samples). However, expression values from normal sample derived from GTEX differed significantly from those derived within TCGA, probably due to the different sample origin and quality. Therefore, only TCGA values are presented within Figure 2 (normal tissue samples: 51, primary tumour samples: 494).

Reference (see references in the Main text)

42. Vivian, J.; Rao, A.A.; Nothaft, F.A.; Ketchum, C.; Armstrong, J.; Novak, A.; Pfeil, J.; Narkizian, J.; Deran, A.D.; Musselman-Brown, A.; et al. Toil enables reproducible, open source, big biomedical data analyses. *Nat. Biotechnol.* **2017**, *35*, 314-316, doi:10.1038/nbt.3772.
43. Goldman, M.J.; Craft, B.; Hastie, M.; Repecka, K.; McDade, F.; Kamath, A.; Banerjee, A.; Luo, Y.; Rogers, D.; Brooks, A.N.; et al. Visualizing and interpreting cancer genomics data via the Xena platform. *Nat. Biotechnol.* **2020**, *38*, 675-678, doi:10.1038/s41587-020-0546-8.
